# The association of childhood adversities and mental health problems with dual-harm in individuals with serious aggressive behaviors

**DOI:** 10.1186/s12888-022-04027-6

**Published:** 2022-06-07

**Authors:** Ying Huang, Simei Zhang, Shaoling Zhong, Ningzhi Gou, Qiaoling Sun, Huijuan Guo, Ruoheng Lin, Weilong Guo, Hui Chen, Jizhi Wang, Jiansong Zhou, Xiaoping Wang

**Affiliations:** 1 Department of Psychiatry, The Second Xiangya Hospital, Central South University, National Clinical Research Center for Mental Disorders, National Technology Institute on Mental Disorders, Hunan Key Laboratory of Psychiatry and Mental Health, Renming Road 139, Changsha, 410011 Hunan Province China; 2 Shenzhen Kangning Hospital, Shenzhen, China; 3 Department of Community Mental Health, The Affiliated Brain Hospital of Guangzhou Medical University, Guangzhou, China

**Keywords:** Dual-harm, Childhood adversities, Mood disorder, Serious aggressive behaviors, China

## Abstract

**Background:**

The coexistence of self-harm and aggression, which is referred to as dual-harm, is commonly seen in forensic population. Self-harm and aggression have often been studied separately, previous studies on risk factors of aggression or self-harm mainly focused on childhood adversities, emotional regulation, impulsivity and psychopathology, given their importance in the two behaviors. However, the factors associated with dual-harm remain unclear. This study aimed to explore potential risk factors associated with co-occurring self-harm among individuals with serious aggressive behaviors.

**Methods:**

This multi-center, cross-sectional case-control study was conducted from May 2013 to January 2016 and involved seven qualified forensic institutes located in seven provinces in China. Participants were individuals with serious aggressive behaviors and were suspected to have mental disorders. Lifetime history of self-harm was obtained by a self-report questionnaire, and serious aggressive behaviors were assessed with the use of participants’ forensic archive. Sociodemographic and clinical information were collected using a self-designed standardized data collection form, and childhood adversities was assessed using a clinician-rated scale designed by our research team. The Psychopathy Checklist-Revised (PCL-R) was used to assess psychopathic traits and the Brief Psychiatric Rating Scale (BPRS) was used to assess psychiatric symptoms of the participants. Univariate and multivariate logistic regression analyses were performed to analyze the relevant factors for dual-harm.

**Results:**

A total of 423 individuals with serious aggressive behaviors were enrolled in the current study. Of them, 74 (17.5%) with self-harm history assigned into the dual-harm group (D-H) and 349 (82.5%) without self-harm history assigned into the aggression-only group (A-O). According to the binary logistic regression analysis, current diagnosis of mood disorder (OR = 3.2, 95%CI: 1.2–8.5), child abuse (OR = 2.8, 95%CI: 1.3–6.2), parental death (OR = 3.0, 95%CI: 1.2–7.5), and the score of the affective subscale in BPRS (OR = 1.7, 95%CI: 1.3–2.4) were significantly associated with dual-harm.

**Conclusions:**

Our study suggested the necessity of integrated evaluation of self-harm among individuals with serious aggressive behaviors. Childhood adversities and psychiatric symptoms in this population require special attention.

**Supplementary Information:**

The online version contains supplementary material available at 10.1186/s12888-022-04027-6.

## Background

Mounting evidence suggested that some individuals engage in both self-harm and aggression during their lifetime [1–3]. The co-existence of self-harm and aggression is termed as dual-harm [4]. A large population-based investigation reported that the prevalence of self-harm co-occurring with aggression (violent crime) was approximately 0.4% [1]. There is an increasing risk of unnatural death and development of riskier patterns of harmful behaviors among dual-harm individuals. In 2019, a study reported that the risk of unnatural death in dual harmers was significantly higher than in the general population or those engaging in aggression towards others or self only [5]. Compared to those who engage in only self-harm or only harm to others, individuals with dual-harm were likely to be different both in quality (e.g., methods of harm) and quantity (e.g., severity and frequency of behavior) [6]. For example, studies showed that dual harmers engaged in more frequent and wider range of harmful behaviors, with increased use of lethal methods [7]. In view of the high-risk behavior pattern of the dual-harm population, intervention is important and warranted for them. Identification of risk factors for dual-harm may help in developing effective prevention strategies for this population.

Individuals engaging in dual-harm are over-represented in forensic and clinical population [7, 8], despite its relatively low incidence in the general population [1]. A study of 326 imprisoned individuals reported that about 42% of the participants had engaged in both aggression and self-harm during their incarceration [3]. Notably, participants in this study were mostly individuals with violent criminal acts, indicating that individuals with serious aggressive behaviors are a high-risk group for dual-harm. However, more attention has been given to these individuals’ aggression towards others instead of harm to themselves [4]. When faced with an individual with serious aggressive behaviors, the usual focus of response is to protect others with the use of punishment, containment or seclusion. However, the punishment-oriented strategies are more likely to increase the risk of future self-harm and antisocial behaviors [9]. Therefore, sufficient awareness of the duality of self-harm and aggression is critical for the successful intervention in individuals with serious aggressive behaviors.

To date, risk factors of dual-harm still remained unclear. Efforts have been put into the investigations on associated factors for dual-harm in the last decade, with special attention to childhood adversities (CAs). Some studies, by distinguishing dual harmers from those who were engaged only in self-harm or aggression towards others, found that childhood adversities might be an independent risk factor for dual-harm [10, 11]. However, previous studies used many different indicators of CAs, such as childhood maltreatment [2], parental death [12], and low family income [13], and it is still unclear which type of CAs, or CAs as a whole, is associated with dual-harm. Another focus of studies on risk factors of dual-harm is mental health problems. Studies have found that many mental disorders are associated with dual-harm, including substance-related disorders, mood disorders, and posttraumatic stress disorder [5, 14]. For instance, Richmond et al. found that dual harmers showed higher comorbidity of substance dependence than single harmers [2]. Steeg et al. found that approximately two thirds of individuals who dual harm were diagnosed with substance use disorders (SUD) [5]. Harford et al. found that dual-harm was closely linked to alcohol/drug use disorder, mood disorders and posttraumatic stress disorder [14]*.* Of note, previous studies have mainly focused on substance-related disorders due to their high incidence in forensic population [15]. However, with more and more studies reporting high levels of other mental disorders in this population [16], it is also important to examine the relationship between dual-harm and other mental disorders in this population. Furthermore, many dimensional measurements of mental health were needed for the exploration of the relationship between dual-harm and mental health, such as the severity of symptoms and the category of diagnosis.

The relationship between personality and self-harm or aggression has been well-established, while its relationship with dual-harm still remains unclear. A previous study revealed that dual harmers were more likely to exhibit personality traits related to emotional and interpersonal liability [2]. Psychopathy is a personality construct marked by interpersonal, affective, and behavioral dysfunction combined with a tendency to express antisocial behaviors [17]. Multiple studies revealed that higher levels of psychopathy was associated with a higher level of delinquent behaviors [18, 19]. Recently, some studies supported that psychopathy, particularly lifestyle-antisocial factors, was associated with suicide-related behaviors [20–23]. There are also studies revealing that some common characteristics of psychopathy, including high impulsivity and high sensation seeking, were linked to serious aggressive behaviors, self-harm and substance-use disorders in the forensic population [24]. Based on the above findings, we speculated that psychopathy might be a potential risk factor of dual-harm.

The concept of dual-harm was proposed in the last few years, and our knowledge about it remains limited. As psychiatric assessments are not routinely performed in individuals with serious aggressive behaviors due to the separation of mental health and justice systems, our knowledge is even more limited about the factors for dual-harm in individuals with serious aggressive behaviors. Based on existing empirical studies, we hypothesized that dual-harm individuals might be impacted by different factors, as compared to those who were engaged in aggression towards self or others only; and the factors for dual-harm might include childhood adversities, history of mental disorders, substance abuse, psychiatric symptoms, and psychopathy. In this case-control study, we aimed to identify associated factors for dual-harm among individuals with serious aggressive behaviors.

## Methods

### Study setting and participants

This study is a multi-center, cross-sectional case-control study started in May 2013 and ended in January 2016. Data were obtained from seven qualified forensic institutes located in seven different provinces in China. Targeted participants were individuals with serious aggressive behaviors and were suspected to have mental disorders. In China, individuals who are engaged in serious aggressive behaviors and suspected to have a mental disorder are required to undergo a forensic psychiatric assessment in order to ascertain their mental status. More details about forensic psychiatric assessment in China can be found in previous literature [25]. The inclusion criteria were individuals who (1) were either female or male, aged over 18, (2) perpetrated any serious aggressive behaviors, including homicide, assault, robbery, arson, and sexual offences, (3) were able to understand and answer the questions in the interview, and (4) were willing to participant in this study. Individuals with incomplete records of the information needed in this study were excluded. The study protocol was approved by the Ethics Committee of the Second Xiangya Hospital. All methods were performed in accordance with relevant guidelines and regulations. Informed consent was obtained from all subjects.

### Definition of dual-harm

Dual-harm was defined as the co-occurrence of self-harm and aggression during an individual’s lifetime. In this study, we defined self-harm as any self-directed injurious behavior with suicidal intent but not resulting in death (i.e., suicide attempt or self-directed injurious behaviors, including poisoning, use of asphyxiating gas, use of sharp objects, hanging, jumping from height, and drowning). Aggression in this study was defined as any serious aggressive behaviors towards others (i.e., violent criminal behaviors), including homicide, assault, robbery, arson, and sexual offences.

### Case and control group

The case group included individuals with both serious aggressive behaviors and a self-harm history, and the control group included individuals with serious aggressive behaviors but without a self-harm history.

### Instruments and evaluation

Lifetime history of self-harm was obtained using a self-report questionnaire, which included questions such as “Have you ever made a suicide attempt, for example, cutting yourself or overdosing on drugs?” Serious aggressive behaviors were ascertained from the participants’ forensic archives in this study. In China, prior to a forensic assessment, the law enforcement agency needs to provide comprehensive relevant information, including demographic information, criminal files (they are collected by the police and include all information on criminal convictions of individuals) and medical records of the individuals engaging in serious aggressive behaviors [26]. These materials, together with the medical reports from the forensic assessment, formed a forensic archive of an individual.

Demographic and clinical information was obtained using a standardized data collection form. The demographic information included gender, age, employment status, marital status, place of residence and education levels, and the clinical information included history of mental disorders, family history of mental disorders, history of head trauma, history of alcohol and non-alcohol substance abuse, previous aggression behaviors, and lifetime treatment for mental disorders; all the above data were also obtained from the participants’ forensic archives. For analysis, the participants’ education levels were categorized into college and above (years of education > 12), middle/high school (years of education ranged 6–12), and primary school and below (illiterate or years of education < 6).

Each participant was evaluated by at least two senior psychiatrists with the use of the International Classification of Diseases 10 (ICD − 10) [27]. Diagnoses of mental disorders were made for participants who meet the corresponding diagnostic criteria. All included diagnoses of mental disorders were classified into five categories: no mental disorder, schizophrenia spectrum disorders, mood disorders, substance-related disorders, and other mental disorders (including personality disorders, neurotic disorders, mental retardation, and other non-specified mental disorders, given the small number of cases).

A clinician-rated scale was used to assess the participants’ childhood adversities. This scale was designed by our research team based on previous studies of violence risks associated with the circumstances in China. This scale is used to assess the adversities before the age of 15 years, including child abuse, parental abandonment, parental death, parental separation, parental psychiatric disorder, parental criminal behaviors and parental alcohol/non-alcohol substance abuse. Information regarding childhood adversities was obtained from participants by asking questions such as “During the first 15 years of your life, have you ever been abused by your parents?” and “Have you lost any of your parents because he/she died?” The total number of childhood adversities that the participant had experienced were recorded and used in subsequent statistical analyses.

The Chinese version of Psychopathy Checklist-Revised (PCL-R) was used to assess psychopathic personality traits in this study [28]. The 20-item PCL-R covered four factors of psychopathy, i.e., interpersonal, affective, lifestyle, and antisocial factors; each item was rated on a 3-point scale (0–2), with the total score ranged 0–40. For this scale, higher scores indicated a higher degree of psychopathy, with a cutoff score of 25 suggesting the presence of psychopathy. This scale has demonstrated satisfactory reliability and validity in previous studies [29].

The 20-item Chinese version of Brief Psychiatric Rating Scale (BPRS) was used to assess psychiatric symptoms in the participants [30]. The BPRS is a well-established clinician-rated tool consisting of 5 subscales: affect (e.g., anxiety, depression), negative symptoms (e.g., withdrawal), positive symptoms (e.g., thought disturbance), activation (e.g., excitement), and resistance (e.g., hostility, suspiciousness). Each item was rated from 1 (no symptom) to 7(extremely severe), and the score of each subscale was calculated by computing the arithmetic mean of the scores of all items included in the subscale.

### Statistical analysis

Categorical variables were presented as number (percentage), and continuous variables were presented as mean ± standard deviation. The missing numerical and categorical values were imputed with median values and modal values, respectively. Categorical variables were analyzed using Chi-square test, and Fisher’s exact test was applied as appropriate. The one-sample Kolmogorov-Smirnov test was used to assess the normality of continuous data. Normally distributed data were analyzed using two-simple t-test and non-normally distributed data were analyzed using the Mann-Whitney U test. Binary logistic regression analysis was performed to investigate the association between dual-harm and other factors, with all the categorical variables set as dummy variables. Before the logistic analysis, we conducted Harman single-factor test to check the common method bias and collinearity statistics for all selected factors. The univariate analysis was first performed to screen potential associated factors, and then the forward stepwise (Likelihood Ratio method) procedures were used to identify independent associated factors for dual-harm. With the type of harm (dual-harm or aggression-only) set as the dependent variable, the factors with *p* < 0.05 in the univariate analysis were selected for the multivariable analysis. All analyses were two-sided, and p < 0.05 were considered as statistically significant. All data were analyzed using the SPSS software (Version 24.0; IBM).

## Results

### Characteristics of the study sample

**Fig. 1:**
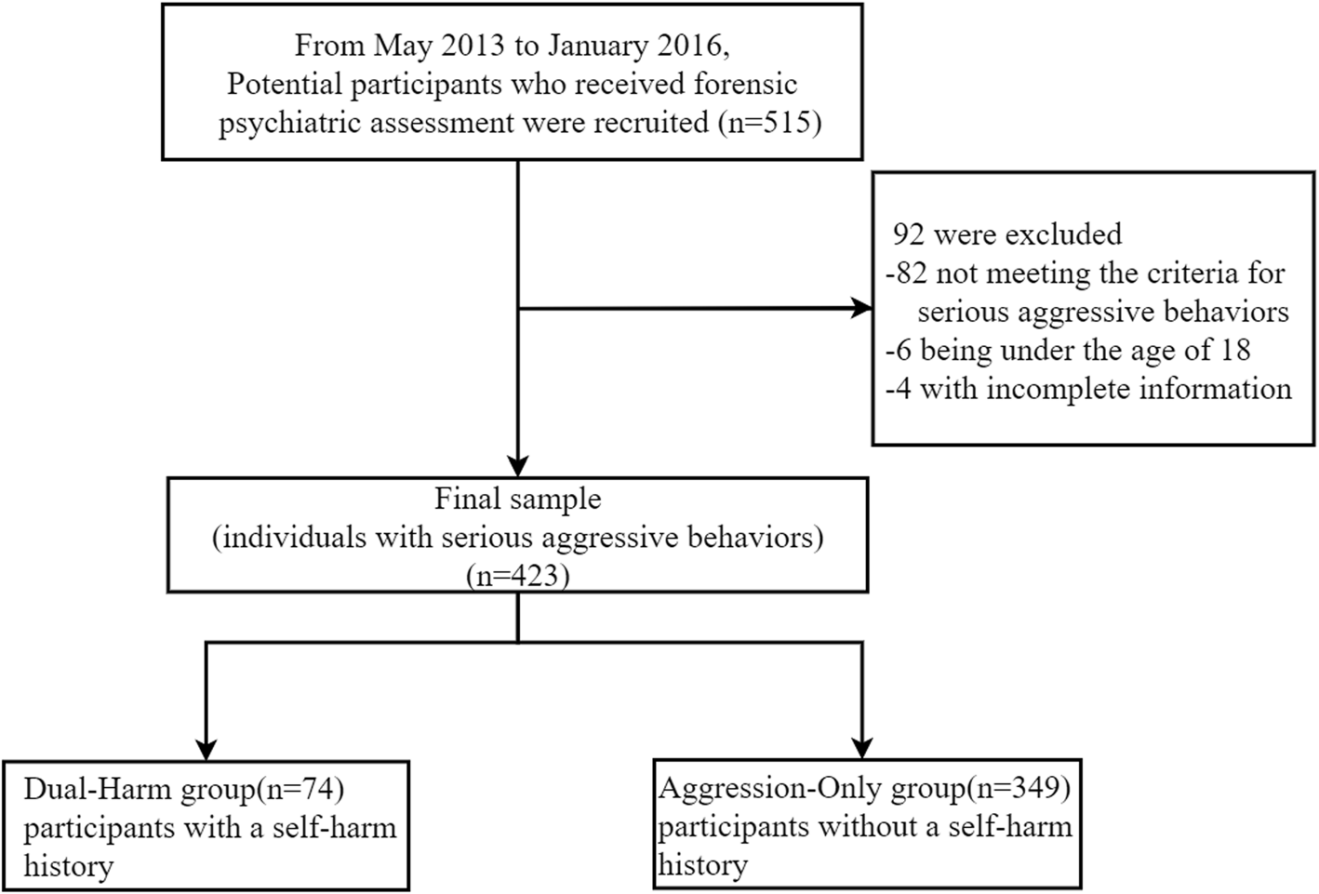
Flow-chart showing sample recruitment

The patient recruitment process is presented in Fig. 1. A total of 515 participants were recruited during the study period. Of them, 82 were excluded for not meeting the criteria for serious aggressive behaviors, 6 were excluded for being under the age of 18 and 4 were excluded for incomplete information. The final sample consisted of 423 participants, with a mean age of 34.25 ± 9.87 years. Seventy-four (17.5%) participants with a self-harm history were assigned into the dual-harm (D-H) group and 349 (82.5%) participants without a self-harm history were assigned into the aggression-only (A-O) group.

### Demographic and clinical characteristics and childhood adversities

A significant difference was found in gender distribution between the two groups (*x*^2^ = 4. 79, *p* = 0.046), and no significant differences were found in other demographic factors, as shown in Table 1.Compared to the A-O group, participants in the D-H group were more likely to have a history of mental disorders (68.9% vs 55.3%, *p* < 0.05). There was no significant difference in other clinical characteristics between the two groups. Among the seven indicators of CAs (see Method), the most prevalent childhood adverse events overall were child abuse (9.5%) and parental separation (9.0%). The D-H group exhibited a higher level of exposure to child abuse and parental death during their childhood, as compared to the A-O group (17.6 and 14.9% vs 7.7 and 4.3%, *p* < 0.05). No significant inter-group difference was found in the other indicators and the total number of CAs (Table 2).

**Table 1:** Demographic characteristics of the D-H and A-O groups

Variable	Total (423)	D-H group	A-O group		
		(74)	(349)	Statistics	*P*
Gender				4.787^a^	0.046
Male	383 (90.5)	62 (83.8)	321 (92.0)		
Female	40 (9.5)	12 (16.2)	28 (8.0)		
Age					
Mean ± SD	34.25 ± 9.87	33.53 ± 9.29	34.40 ± 10.00	0.689^b^	0.491
Unemployment	220(52.0)	33 (44.6)	187 (53.6)	1.976^a^	0.200
Marital status				2.565^c^	0.442
Never married	233 (55.1)	44 (59.5)	189(54.1)		
Married	124(29.3)	17 (23.0)	107 (30.7)		
Divorced	59(13.9)	11 (14.9)	48(13.8)		
Widowed	7(1.7)	2 (2.7)	5(1.4)		
Residence				1.635^a^	0.249
Urban	200(47.3)	30 (40.5)	170(48.7)		
Rural	223 (52.7)	44 (59.5)	179 (51.3)		
Education^1^				0.810^c^	0.670
Primary school and below	277 (65.5)	52(70.3)	225 (64.5)		
Middle/high school	126(29.8)	19(25.7)	107 (30.7)		
College and above	20 (4.7)	3(4.1)	17(4.8)		

Education^1^: Primary school and below: ≤ 6 years of education, Middle/high school: 6–12 years of education, College and above: > 12 years of education. ^a^: Chi-squared test, ^b^: Two samples t test, ^c^: Fisher’s exact test. *D-H* dual-harm, *A-O* aggression-only

**Table 2:** Clinical characteristics and childhood adversities of the D-H and A-O groups

Variable	Total (423)	D-H group	A-O group		
		(74)	(349)	Statistics	*P*
History of mental disorders, n (%)	244 (57.7)	51 (68.9)	193 (55.3)	4.639^a^	0.038
Family history of mental disorders, n (%)	68(16.1)	16 (21.6)	52(14.9)	2.045^a^	0.164
History of head trauma, n (%)	42 (9.9)	12(16.2)	30 (8.6)	3.964^a^	0.055
History of alcohol abuse, n (%)	65(15.4)	8 (10.8)	57 (16.3)	1.431^a^	0.288
History of non-alcohol substance abuse, n (%)	45 (10.6)	13 (17.6)	32 (9.2)	4.530^a^	0.039
Previous aggression history, n (%)	371 (87.7)	67(90.5)	304 (87.1)	0.668^a^	0.447
Treatment experience				1.572^c^	0.462
Untreated	160 (37.8)	24 (32.4)	136 (39.0)		
Outpatient	90 (21.3)	19 (25.7)	71 (20.3)		
Inpatient	173 (40.9)	31 (41.9)	142 (40.7)		
Indicators of childhood adversity					
Child abuse, n (%)	40 (9.5)	13 (17.6)	27 (7.7)	6.892^a^	0.012
Parental abandonment, n (%)	15 (3.5)	3 (4.1)	12 (3.4)	0.068^a^	1.000
Parental death (Loss of one/both parents)	26 (6.1)	11 (14.9)	15 (4.3)	11.818^a^	0.002
Parental separation	38 (9.0)	4 (5.4)	34(9.7)	1.404^a^	0.273
Parental psychiatric disorder	7 (1.7)	3 (4.1)	4 (1.1)	3.172^a^	0.106
Parental criminal behavior	7 (1.7)	3 (4.1)	4 (1.1)	3.172^a^	0.106
Parental alcohol/substance abuse	17 (4.0)	5 (6.8)	12 (3.4)	1.743^a^	0.194
Number of indicators of childhood adversity				10.544^c^	0.025
0	277 (65.5)	42 (56.7)	235 (67.4)		
1	102 (24.1)	20 (27)	82 (23.5)		
2	35 (8.3)	7 (9.5)	28 (8.0)		
≥3	9 (2.1)	5 (6.8)	4 (1.1)		

^a^: Chi-squared test, ^c^: Fisher’s exact test. *D-H* dual-harm, *A-O* aggression-only

### Current psychiatric diagnoses

Overall, there were 354 (83.7%) participants diagnosed with mental disorders, with the most common diagnoses being schizophrenia spectrum disorders, followed by mood disorders and substance-related disorders. The proportion of participants diagnosed with mood disorders and substance-related disorders was higher in the D-H group than in the A-O group (20.3 and 12.2% vs 6.3 and 7.7%), and the proportion of participants diagnosed with schizophrenia spectrum disorders and other mental disorders were lower in the D-H group than in the A-O group (48.6 and 4.1% vs 57.6 and 11.7%). Compared to those in the A-O group, participants in the D-H group had a higher score of the anti-social subscale (T = -2.29, *p* = 0.02), as well as a higher score of the affective subscale in BPRS (T = -3.42, *p* = 0.001) (Table 3).

**Table 3:** Current psychiatric diagnoses and assessments in the D-H and A-O groups

Variable	Total (423)	D-H group	A-O group		
		(74)	(349)	Statistics	P
Current diagnoses				17.296^c^	0.001
Non-psychiatry	69 (16.3)	11 (14.9)	58 (16.6)		
Schizophrenia spectrum disorders	237 (56.0)	36 (48.6)	201 (57.6)		
Mood disorders	37 (8.7)	15 (20.3)	22 (6.3)		
Substance-related disorders	36 (8.5)	9 (12.2)	27 (7.7)		
Other	44 (10.4)	3 (4.1)	41 (11.7)		
PCL-R					
Total		14.38 ± 9.82	12.06 ± 7.99	-1.90^b^	0.061
Interpersonal		1.86 ± 2.45	1.66 ± 1.91	-0.79^b^	0.431
Emotion		2.78 ± 2.25	2.72 ± 2.51	-0.20^b^	0.843
Lifestyle		5.26 ± 3.02	4.50 ± 3.24	-1.87^b^	0.063
Anti-social		3.55 ± 2.78	2.77 ± 2.12	-2.29^b^	0.024
BPRS					
Anxiety-depression		2.16 ± 0.89	1.79 ± 0.85	-3.42^b^	0.001
Withdrawal		2.06 ± 1.05	2.13 ± 1.04	0.52^b^	0.603
Thought disturbance		2.63 ± 2.05	2.63 ± 1.32	0.001^b^	0.999
Activation		1.87 ± 0.89	1.84 ± 1.06	-0.28^b^	0.778
Hostile suspicion		2.89 ± 1.49	3.04 ± 1.71	0.80^b^	0.426

^b^: Two simple t-test, ^c^: Fisher’s exact test. *D-H* dual-harm, *A-O* aggression-only, *PCL-R* Psychopathy Checklist- Revised, *BPRS* Brief Psychiatric Rating Scale

### Associated factors for dual-harm

The Harman’s single-factor test result (23.6%), which was less than the critical value (40%), indicated no serious common method bias in the present study. Results of collinearity statistics showed that the VIF of candidate predictors ranged from 1.025 to 1.883, indicating no collinearity. In the final regression model, current diagnoses of mood disorders (OR = 3.2, 95%CI: 1.2–8.5), child abuse (OR = 2.8, 95%CI: 1.3–6.2), parental death (OR = 3.0, 95%CI: 1.2–7.5), and the score of the affective subscale in BPRS (OR = 1.7, 95%CI: 1.3–2.4) were independently associated with increased risk of dual-harm in individuals with serious aggressive behaviors. The Hosmer-Lemeshow test values were reported at χ2 = 6.70, *p* = 0.57, while the Nagelkerke R Square value was 0.16. The results are presented in Table 4.

**Table 4:** Binary logistic regression analysis for dual-harm in individuals with serious aggressive behaviors(n, %)

Variable	D-H group (74)	A-O group (349)	Univariate analysis OR (95% CI)	*p*-value	Multivariate analysis OR (95% CI)	*p*-value
Gender (female)	12 (16.2)	28 (8.0)	2.2(1.1–4.6)	0.032	NI	
History of mental disorders	51 (68.9)	193 (55.3)	1.8(1.0–3.1)	0.033	NI	
History of non-alcohol substance abuse	13 (17.6)	32 (9.2)	2.0(1.0–4.3)	0.037	NI	
Child abuse	13 (17.6)	27 (7.7)	2.5(1.2–5.2)	0.011	2.8(1.3–6.2)	0.01
Parental death	11 (14.9)	15 (4.3)	3.9(1.7–8.9)	0.001	3.0(1.2–7.5)	0.02
Number of indicators of childhood adversity(1)	20(27)	82(23.5)	1.3(0.8–2.5)	0.301	NI	
Number of indicators of childhood adversity(2)	7(9.5)	28(8.0)	1.4(0.6–3.4)	0.460	NI	
Number of indicators of childhood adversity(≥3)	5(6.8)	4(1.1)	7.0(1.8–27.1)	0.005	NI	
Current diagnoses of mood disorders	36(48.6)	201(57.6)	3.6(1.4–9.0)	0.006	3.2(1.2–8.5)	0.02
Current diagnoses of schizophrenia spectrum disorders	15(20.3)	22(6.3)	0.9(0.5–2.0)	0.879	0.7(0.3–1.6)	0.44
Current diagnoses of substance-related disorders	9(12.2)	27(7.7)	1.8(0.7–4.7)	0.265	1.4(0.5–3.9)	0.55
Current diagnoses of other mental disorders	3(4.1)	41(11.7)	0.4(0.1–1.5)	0.163	0.3(0.1–1.3)	0.10
Score of the affective subscale in BPRS	2.16 ± 0.89	1.79 ± 0.85	1.6(1.2–2.1)	0.001	1.7(1.3–2.4)	< 0.001
Score of the anti-social subscale in PCL-R	3.55 ± 2.78	2.77 ± 2.12	1.2(1.0–1.3)	0.007	NI	

*NI* not included in the final model; *D-H* dual-harm, *A-O* aggression-only; *PCL-R* Psychopathy Checklist- Revised; *BPRS* Brief Psychiatric Rating Scale

## Discussion

To our knowledge, this is the first study to investigate the independent factors for dual-harm among individuals with serious aggressive behaviors in China. In the present study, we used a case-control study design and found that current diagnoses of mood disorders, child abuse, parental death and the score of the affective subscale in BPRS were significantly associated with dual-harm among individuals with serious aggressive behaviors.

Childhood adversities may contribute to the development of many maladaptive behaviors, including aggression, self-harm, and violence [31, 32]. Our findings suggested that dual harmers had higher levels of exposure to child abuse and parental death during their childhood, as compared with the individuals engaging in aggression towards others only. This was consistent with previously reported findings; for example, Richmond et al. found that dual harmers exhibited more experience of childhood maltreatment than those engaging in aggression towards others [2]. A prior study found that parental death was associated with increased risk of both self-harm and serious aggressive behaviors (violent crime) [12], and some researchers found that the risk of dual-harm among individuals who experienced parental death was even greater [10]. However, inconsistent with previous studies, we did not find the association between the total number of CAs and dual-harm. A longitudinal cohort study, which used nearly the same indicators of CAs as those in our study, revealed that individuals with a history of violent offenses who had also experienced cumulative childhood adversities had a higher risk of suicide [11]. Another study using different indicators of CAs found that the experience of five or more childhood adverse events was more frequently observed in dual harmers [10]. Our findings suggested that the type of CAs, instead of the number of CAs, was associated with dual-harm. This was not unexpected, since child abuse was believed to be associated with long-term emotional and physical health problems [33], and parental death was regarded as the most stressful event during an individual’s upbringing and may lead to lasting adverse consequences [34]. It is also important to note that the construct of CAs in our study was not exactly the same as the construct of Adverse Childhood Experiences (ACEs), which was dominant in the literature [35]. The World Health Organization (WHO) defines ACEs as a broad set of negative childhood experiences, including abuse (physical, emotional, or sexual) and neglect (physical or emotional), serious household dysfunction (e.g., witnessing domestic violence, drug use of a household member, parental separation and incarceration), and peer, community, and collective violence [36]. The measures of CAs included in the present study is relatively narrow, capturing only information of abuse and serious household dysfunction while excluding information of physical or emotional neglect, peer, community, and collective violence. The relationship between the unexplored childhood adversities and dual-harm will be investigated in the future. In addition, the prevalence of CAs in our study is remarkably low compared to other studies within China and abroad. For example, the prevalence of parental separation was 9.0% in our study, apparently lower than 20% in another domestic study [37]. Furthermore, in our study, the proportion of participants who reported at least one CA was 24.1%, lower than that reported in Danish and Sweden (27.5 and 32.5%, respectively [10, 11]. This result might be attributed to the potential information bias. First, the participants might have difficulty recalling their childhood adversities as they happened a long time ago; second, some individuals might be reluctant to report, or even recall such events; third, concepts such as child abuse and child neglect were not given importance in earlier years.

The relationship between mental disorders and dual-harm has not been adequately studied. Our study found that dual harmers were more likely to be diagnosed with mood disorders compared to those who only engaged in aggression towards others, which was in consistent with the previous findings. Harford et al. reported that dual-harm was more strongly associated with mood disorders [14]. Furthermore, it has been reported that depression is the most common diagnosis in individuals who engaged in murder-suicide, an extreme dual-harm behavior [38]. The above results suggested that individuals with mood disorders needed special attention in studies on dual-harm. Other mental disorders should not be ignored either. A previous study showed that dual harmer had higher rates of psychiatric comorbidity and were faced with more difficulties, but they might not be able to receive additional mental health services [2], indicating that more attention should be given to the comorbidity of mental disorders in dual-harm individuals. Nevertheless, we did not find the association between dual-harm and substance-related disorders (including history of substance abuse and current diagnoses of substance-related disorders), which was considered to be a major risk factor for dual-harm in western countries [14, 39, 40]. The results might be explained with the following reasons. For one hand, the patterns of substance abuse differed greatly between different regions. For example, studies in Asian countries reported lower rates of comorbidity of substance abuse among individuals with mental disorders, as compared with those in western countries [41]. The relatively low incidence of substance-related disorders in our sample might have contributed to the difficulty in identifying the association. On the other hand, researches revealed that drug users and individuals with serious aggressive behaviors might share some characteristics and personalities (such as high impulsivity and high sensation seeking), which place them at a higher risk of both aggression and drug use; these characteristics were also high-risk factors for self-harm. The shared characteristics might be a reason for the similar incidences of substance-related disorders in the dual-harm group and the aggression-only group.

Another finding in the present study was that the dual-harm group scored higher on the affective subscale in BPRS than the aggression-only group, and this difference persisted after accounting for known risk factors. Few studies have focused on psychiatric symptoms in dual harmers, and only one study reported that individuals with dual-harm ideation had higher scores related to depression than those who had aggression ideation only [42]. This result could be explained from the following perspectives. From the psychodynamic aspect, individuals with depression tend to use the defense mechanism of introjection, leading to aggression towards themselves [43]. In addition, some empirical studies revealed that anxiety and depressive symptoms were strongly related to self-oriented aggression, rather than aggression towards others [44, 45]. Finally, according to the theories about dual-harm, depression might lead to aggression towards self [46].

Some researchers proposed that a personality characterized by a predisposition to engage in harmful behaviors might be shaped by biological factors interacting with adverse early environments [47]; they emphasized that the secondary psychopathy characterized by antisocial and unstable lifestyle could be a risk factor for dual-harm [47]. In our study, the dual-harm group scored higher on the antisocial scale than the aggression-only group, which supported the previous views. However, this variable was not entered into the final regression model. Our results do not seem to support the association between psychopathy and dual-harm. This is understandable, as evidence on the relationship between psychopathy and self-harm varied greatly. For example, Cleckley suggested that ‘less suicidal behavior’ is a major characteristic of psychopathy [48]. However, Verona found that some factors of psychopathy was significantly associated with suicide attempts [49]. As the relationship between psychopathy and self-harm is unclear, the relationship between psychopathy and dual-harm remains much more uncertain. However, given some other empirical evidence supporting psychopathy as a key factor for self-harm and aggression [21, 50], the role of psychopathy in dual-harm cannot be neglected. The present results could be attributed to the methodology, since some studies revealed a significant association between psychopathy and self-harm when self-rated scales were used. Therefore, methods for the measurement of psychopathy need to be carefully selected in future studies.

Despite the strength of our study, some limitations should also be noted. First, participants in our study were individuals with serious aggressive behaviors and underwent forensic psychiatric assessments, thus, the extrapolation of our findings to other populations needs further validation. Second, the definition of self-harm in this study was limited to suicide attempt, whereas aggression was limited to serious aggressive behaviors (violent criminal behaviors); the relatively narrow scope of the definitions might have led to underestimation of the rate of dual-harm in the selected population. Third, another limitation of the present study is that we did not explore factors of dual-harm in males and females separately due to the relatively small number of females, thus, gender might still be a confounding factor. Fourth, an important methodological limitation is that we did not use a validated scale for CAs assessment, which may impact on the generalizability of our findings. Fifth, a self-reported scale was used to obtain self-harm information from the participants, which might lead to memory biases. Sixth, as this is a cross-sectional study, the causal relationship could not be established. Seventh, comorbidities of mental disorders were not taken into account, as only the principal diagnosis was recorded in some patient files. Eighth, due to the retrospective nature of data collection, some potentially important factors, such as emotional regulation, impulsivity, neuroticism, childhood neglect, peer, community, and collective violence, could not be included in this study. These factors need to be considered in future studies.

## Conclusions

Although some factors for dual-harm have been identified, this phenomenon has not been fully understood. In the present study, we investigated several factors known to be associated with dual-harm, such as childhood adversities, substance abuse, and history of mental disorders, and explored some potentially associated factors, such as psychopathology and psychopathy. We found that substance abuse among individuals with serious aggressive behaviors in China are different, to some extent, from those observed in other countries. Our findings suggested that a comprehensive assessment and detailed evaluation of self-harm is needed for individuals with serious aggressive behaviors, which calls for the collaborative effort of justice systems and the mental health sector. The identified risk factors suggest that effective strategies for the prevention of dual-harm among individuals with serious aggressive behaviors should include the strengthening of mental health services for this population and early intervention for children who have adverse experiences. In the future, a longitudinal design should be considered to examine the causal relationship between those variables. Future studies are also needed to explore the risk factors of dual-harm in different countries and under different jurisdictions.

## Supplementary Information

**Additional file 1.**

**Additional file 2.**

## Data Availability

The data generated or analyzed during this study are included in this manuscript and its Supplementary materials.

## Abbreviations

A-O: Aggression-only
ACEs: Adverse Childhood Experience
BPRS: Brief Psychiatric Rating Scale
CAs: Childhood Adversities
D-H: Dual-harm
PCL-R: Psychopathy Checklist- Revised
SUD: Substance use disorders
